# Genetic and particle modelling approaches to assessing population connectivity in a deep sea lobster

**DOI:** 10.1038/s41598-022-19790-5

**Published:** 2022-10-06

**Authors:** Aimee L. van der Reis, Craig R. Norrie, Andrew G. Jeffs, Shane D. Lavery, Emma L. Carroll

**Affiliations:** 1 Institute of Marine Science, University of Auckland, Auckland, New Zealand; 2 School of Aquatic and Fisheries Sciences, University of Washington, Seattle, USA; 3 School of Biological Sciences, University of Auckland, Auckland, New Zealand

**Keywords:** Ecology, Genetics

## Abstract

The emergence of high resolution population genetic techniques, such as genotyping-by-sequencing (GBS), in combination with recent advances in particle modelling of larval dispersal in marine organisms, can deliver powerful new insights to support fisheries conservation and management. In this study, we used this combination to investigate the population connectivity of a commercial deep sea lobster species, the New Zealand scampi, *Metanephrops challengeri*, which ranges across a vast area of seafloor around New Zealand. This species has limited dispersal capabilities, including larvae with weak swimming abilities and short pelagic duration, while the reptant juvenile/adult stages of the lifecycle are obligate burrow dwellers with limited home ranges. Ninety-one individuals, collected from five scampi fishery management areas around New Zealand, were genotyped using GBS. Using 983 haplotypic genomic loci, three genetically distinct groups were identified: eastern, southern and western. These groups showed significant genetic differentiation with clear source-sink dynamics. The direction of gene flow inferred from the genomic data largely reflected the hydrodynamic particle modelling of ocean current flow around New Zealand. The modelled dispersal during pelagic larval phase highlights the strong connectivity among eastern sampling locations and explains the low genetic differentiation detected among these sampled areas. Our results highlight the value of using a transdisciplinary approach in the inference of connectivity among populations for informing conservation and fishery management.

## Introduction

Deep sea species have increasingly become commercial targets due to improving fishing technology and an increasing global demand for seafood that is unfulfilled by declining catches from shallower coastal waters^1–4^. However, our knowledge of the biology of many of these deep sea species is poor due to the significant challenges in undertaking research in the deep sea environment, however, experience indicates these populations are vulnerable to overfishing^5^. Thus, it is critical to gain insight into these patterns of population connectivity in these targeted species to adopt best practice fisheries management approaches that nurture stock and the environment sustainably in the deep sea. Furthermore, deep sea species frequently have distribution ranges that cover vast areas, and are frequently patchily distributed throughout those areas. This creates potential challenges for maintaining genetic connectivity of the population, especially where the dispersal capacity of the species may be limited. In such instances, broad scale hydrodynamic processes, such as major current systems, can be expected to play a vital role in maintaining connectivity within the broad scale of the population range^6^.

Patterns of genetic diversity and connectivity are not well understood in deep sea invertebrate species, in stark contrast to the comparative large body of knowledge on their shallow-water and terrestrial counterparts^7^. Characterising patterns of genetic population structure in part of a species range provides insight into levels and directions of gene flow that could be extrapolated across its distribution, and be useful for fisheries management, such as for designating fishery management areas (FMAs). It can also provide essential information for understanding the drivers of neutral and adaptive genetic diversity within exploited populations^8–15^.

In general, the loss of genetic diversity has the potential to reduce productivity, population persistence and adaptability, posing the risk of increasing the vulnerability of species already under commercial fishing pressure^16,17^. Thus, determining source-sink dynamics, including larval dispersal and gene flow patterns, has direct ecological implications for the persistence and structure of populations of deep sea animals that are commercially harvested^6^. Here we employ the definition that a source is a population in which the net export of individuals or genetic material is greater than the net import, while the reverse is a sink^6^. A common feature of sink populations, other than their genetic landscape being shaped to a degree by receiving gene flow via larval arrivals from multiple sources, is a tendency for increased levels of genetic diversity^18^. However, other demographic factors may also contribute to maintaining genetic diversity in a population, such as large local population size^19^. Thus, it is also possible for large source populations to maintain relatively high levels of genetic diversity, despite elevated levels of self-recruitment.

The results of many marine population genetic studies suggest that larval dispersal, prevailing currents and geographic distance are all important factors that have been implicated in determining patterns of connectivity^20–23^. In particular, several studies have highlighted the existence of asymmetric patterns of connectivity, revealing source-sink relationships largely determined by currents^24,25^. Such relationships are most likely to be observed in species with limited adult and larval dispersal. Arguably, deep sea species with these traits offer an excellent opportunity for evaluating the efficacy of combining population genomics with the modelling of physical dispersal processes that could be expected to be the dominant process for structuring the population, having had less long term anthropogenic disturbance.

**Figure 1:**
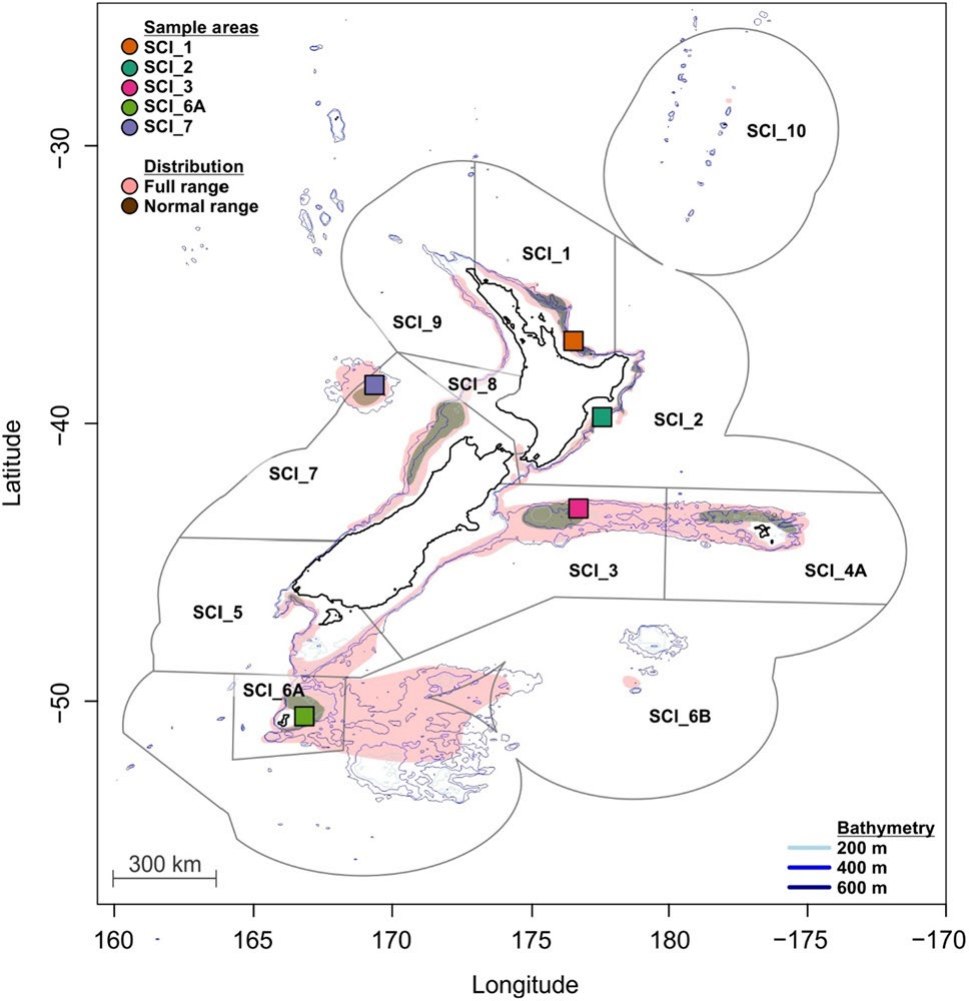
Map of the scampi sampling locations (squares) with bathymetry shown to 600 m in 200 m increments (MARMAP v1.3.4^113^). Scampi were collected from five scampi commercial fishery management areas: SCI_1 (Bay of Plenty), SCI_2 (Hawke’s Bay and Wairarapa Coast), SCI_3 (Mernoo Bank, on the Chatham Rise), SCI_6A (Sub-Antarctic) and SCI_7 (West Coast). Indicated on the map is the sampling area within each SCI (coloured square) and the approximate normal (90% distribution) and full scampi range^28^. The normal distribution is largely targeted by commercial fisheries and is heavily fished. Coordinates of trawls and sample area corners are provided in Table S1. The figure's main elements, i.e., New Zealand, sample areas and particle routes, were generated with ggplot2 v3.3.3^114^, mapdata v2.3.0^115^ and mapproj v1.2.7^116^. Other aesthetic elements were added using Corel Draw Essentials X5 (v15.2.0.686).

The New Zealand scampi (*Metanephrops challengeri*), hereafter referred to as scampi, is a deep sea endemic lobster species. Scampi are fished by commercial bottom trawling over extensive areas of the continental shelf of New Zealand below 100 m depth, including around offshore islands^26^. The fishery is divided into 10 spatial management areas (officially referred to as SCI—scampi fishery management area; i.e., SCI1-10) based only on arbitrary geographic boundaries (Fig. 1) and not any meaningful population metrics, thereby creating uncertainties in fisheries management within these areas^27^. The scampi population has a vast range in the waters around New Zealand (Fig. 1). Extensive prospecting by commercial fishers identifying and focusing harvesting attention on those areas where the species is found in higher densities (i.e., 90% distribution described as the normal range; Fig. 1), especially deep sea seafloor plateaus, indicates that the distribution of the population is not spatially homogenous over its range^28^. Two of the SCIs are of greatest fisheries importance; SCI_3 and SCI_6A, which together account for 54% of the total allowable commercial catch for the species, with annual landings in these areas of 408 t and 306 t, respectively^26^. The larval dispersal distance of scampi is likely constrained because of an abbreviated pelagic larval duration (PLD) of typically less than 11 days, and their very poor swimming abilities throughout the larval stages^29,30^. Despite intensive commercial fishing of this species, relatively little else is known about their biology (including their growth) and stock structure. Juveniles and adults of scampi occupy burrows in soft benthic sediment and emerge to scavenge for food in the immediate vicinity of their burrows^31–33^. Therefore, it is highly unlikely that the movement of the reptant phase in the lifecycle of scampi is the primary contributor to gene flow within the population range. Given these biological constraints on the extent of gene flow, we predict that there is a relatively high degree of genetic structure within the New Zealand-wide distribution of this species.

A transdisciplinary method of understanding the patterns of population connectivity among stocks is to combine population genetic analyses, using next-generation DNA sequencing methods, with estimates of animal dispersal using physical modelling of particles^25,34–40^. Genetic data can provide estimates of population connectivity through genetic differentiation and inferred directions of gene flow through migration rates, while particle modelling can provide simulations of the direction of movement during the pelagic larval phase. The directionality and coherence of currents can indicate the potential routes of pelagic larval dispersal over multiple generations, assuming the availability of intermediate habitat for larvae to settle, establish and reproduce. This general approach of investigating genetic data by integrating environmental parameters to evaluate their potential influence on population genetic structure is coined seascape genetics^23^ and has increased in popularity as a method to study population connectivity over the past 10 years, although still limited to less than 100 publications^41^. For marine species which rely on larval dispersal, ocean hydrodynamics are likely to play the predominant role in determining dispersal trajectories in space and time, and this hypothesis can be tested by comparing genetic inferences about connectivity with estimates of connectivity driven by hydrodynamics. Understanding patterns of genetic diversity and population connectivity is essential information that is often lacking for fisheries management and conservation in species harvested from across vast population ranges, such as scampi^42,43^. This approach helps to elucidate the role of predominating ocean currents and their relationship in driving the observed genetic and distribution patterns of species (e.g., deep sea shrimp^22^ and limpets^44^). Here we examine the population connectivity of the New Zealand scampi by combining genotyping-by-sequencing (GBS) analysis and particle modelling methods to better understand this deep sea invertebrate’s genetic connectivity. Although the known population range of this species is vast, the distribution density is concentrated to certain known areas coinciding with the most heavily fished areas, which are geographically dispersed. We expect that the limited larval dispersal abilities of this species could have constrained connectivity among fishery management areas, with prevailing ocean currents driving the development of different source-sink relationships within the broadly distributed scampi population. The connectivity estimates from the genetic data (e.g., genetic differentiation and migration rates) will be compared to connectivity estimates from the modelling data (e.g., patterns of movements during the pelagic larval phase and likely routes of travel in subsequent generations). This will allow us to infer connectivity among these dispersed locations and potential source-sink dynamics. We aim to improve our understanding of the resilience of the deep sea scampi population to commercial fishing and contribute towards future management of this fishery.

## Results

### Initial data quality control

A total of 289,502,412 raw reads were analysed for quality control using FASTQC (all software/R packages are denoted similarly; Table S2). Their sequence length varied from 35 to 101 base pairs (bp) and GC content was 52%. Per base sequence quality scores dropped with read length over 75 bp. PROCESS RADTAGS retained 244,889,406 reads which had barcodes and cut-sites intact, and were above the minimum quality threshold.

### De novo genotyping and data filtering

After USTACKS was run, 162,546,729 (56.1%) reads remained after having been aligned into stacks (Table S2). The CSTACKS catalogue contained 126,822 loci of which GSTACKS genotyped 123,778 (97.6%). Six poor performing individuals were removed from subsequent analyses, three from both SCI_3 (individuals 247.9, 257.5 and 257.8) and SCI_7 (WC1.7, WC1.12 and WC2_rep1a). The large drop in number of loci from the initial catalogue to the final haplotypic dataset is due to the conservative measures (i.e., all loci present in > 80% of individuals and in each population; POPULATIONS) taken to ensure a high quality dataset that would provide reliable results, and not biased by missing data. Quality control (QC) replicates (WC2_rep2) showed 98% similarity and WC2_rep2b, which had slightly lower quality, was excluded from further analyses. QC further eliminated 20 loci that had sequencing depth > 2 × the standard deviation (SD) above the mean locus depth, and 51 loci with > 5 single nucleotide polymorphisms (SNPs) per locus, thereby removing potential repetitive regions and paralogues.

The final haplotypic dataset had a total of 983 loci containing a total of 2,115 SNPs, across 86 individuals that passed QC. The average proportion of missing data per individual and per SNP site was 0.03 (SD = 0.04) and 0.03 (SD = 0.04), respectively (Tables S3 and S4). Loci had on average 2.15 SNPs (SD = 1.16), with an average MAF per variable site of 0.07 (SD = 0.11; Table S5), and were typed to a mean depth per locus per individual of 366.97 reads (SD = 225.30; Table S6).

Only four outliers were detected and only one was identified by all analyses (Table S7). No outlier DNA sequences were confidently matched to any GenBank sequences. Due to the very weak evidence for selection in the genetic data set, the outlier loci were not analysed separately.

### Patterns of genetic diversity and differentiation

#### Genetic diversity and differentiation

Of the sampled fishery management areas, SCI_3 had the greatest mean observed (H_o_) and expected (H_e_; gene diversity) heterozygosity, allelic richness (A_r_) and nucleotide diversity (π) and the lowest inbreeding coefficient (F_IS_) (Table 1). SCI_7 had the lowest mean H_o_, H_e_, A_r_ and π. In general, similar values were seen among SCI_1, SCI_2 and SCI_3 and between SCI_6A and SCI_7 for the diversity measures. However, F_IS_ showed a decrease from SCI_1 to SCI_3, while within SCI_6A and SCI_7 they were similarly high.

**Table 1:** Summary statistics for each scampi fishery management area (SCI) based on the mean values across 983 loci.

SCI	n	A_r_	F_IS_	H_o_	H_e_	π
1	18	1.658 (0.417)	0.042 (0.241)	0.167 (0.182)	0.177 (0.180)	0.099 (0.144)
2	18	1.652 (0.422)	0.035 (0.237)	0.169 (0.183)	0.176 (0.178)	0.098 (0.144)
3	15	1.671 (0.441)	0.024 (0.241)	0.176 (0.190)	0.181 (0.181)	0.101 (0.146)
E	51	1.660 (0.427)	0.034 (0.234)	0.170 (0.185)	0.178 (0.180)	0.099 (0.144)
6A	17	1.569 (0.463)	0.040 (0.247)	0.154 (0.187)	0.163 (0.182)	0.092 (0.150)
7	18	1.553 (0.461)	0.038 (0.252)	0.154 (0.192)	0.161 (0.185)	0.091 (0.150)

SCI_E is composed of SCI_1, SCI_2 and SCI_3. Standard deviation is enclosed in brackets. SCI, scampi fishery management area; n, number of individuals; A_r_, allelic richness; F_IS_, inbreeding coefficient; H_o_, observed heterozygosity; H_e_, expected heterozygosity; π, nucleotide diversity.

Among all SCIs there was significant genetic differentiation as measured by the F_ST_ value of 0.036 (*p* value < 0.001). Pairwise comparisons of SCIs based on F_ST_ values showed that the five SCIs were grouped into three genetic clusters (Table 2). There was no significant difference among the SCI_1, SCI_2 and SCI_3 (SCIs that are predominantly east of the main islands of New Zealand; collectively named ‘SCI_E’), but there was significant genetic differentiation for all other pairwise combinations, i.e., among SCI_E, SCI_6A and SCI_7.

**Table 2:** Pairwise F_ST_ between scampi fishery management areas (SCIs) (lower diagonal) and Bonferroni adjusted *p* values (upper diagonal).

	SCI_1	SCI_2	SCI_3	SCI_6A	SCI_7	SCI_E
SCI_1		0.740	1.000	< 0.001	< 0.001	–
SCI_2	0.003		1.000	< 0.001	< 0.001	–
SCI_3	0.002	0.002		< 0.001	< 0.001	–
SCI_6A	0.059*	0.055*	0.051*		< 0.001	< 0.001
SCI_7	0.057*	0.057*	0.048*	0.020*		< 0.001
SCI_E	–	–	–	0.052*	0.051*	

SCI_E is composed of SCI_1, SCI_2 and SCI_3. F_ST_ values that were found to be significant (*p* value < 0.05) are marked (*).

The AMOVA results complemented the pairwise results, as significant differences were found among all sample partitions analysed except those within SCI_E (Table 3). More than 90% of variance was found within SCIs rather than between (i.e., Φ_ST_ < 0.10).

**Table 3:** Analysis of molecular variance (AMOVA) between scampi fishery management areas (SCIs) based on 983 loci.

	Φ_ST_	*p* value
All SCIs	0.067*	< 0.001
SCI_1 vs 2 vs 3	0.003	0.135
SCI_6A vs 7	0.039*	< 0.001
SCI_E vs 6A	0.096*	< 0.001
SCI_E vs 7	0.096*	< 0.001
SCI_E vs 6A vs 7	0.089*	< 0.001

SCI_E is composed of SCI_1, SCI_2 and SCI_3. Φ_ST_ (Phi-_ST_ statistic) values that were found to be significant (*p* value < 0.05) are marked (*).

#### Genetic clustering

**Figure 2:**
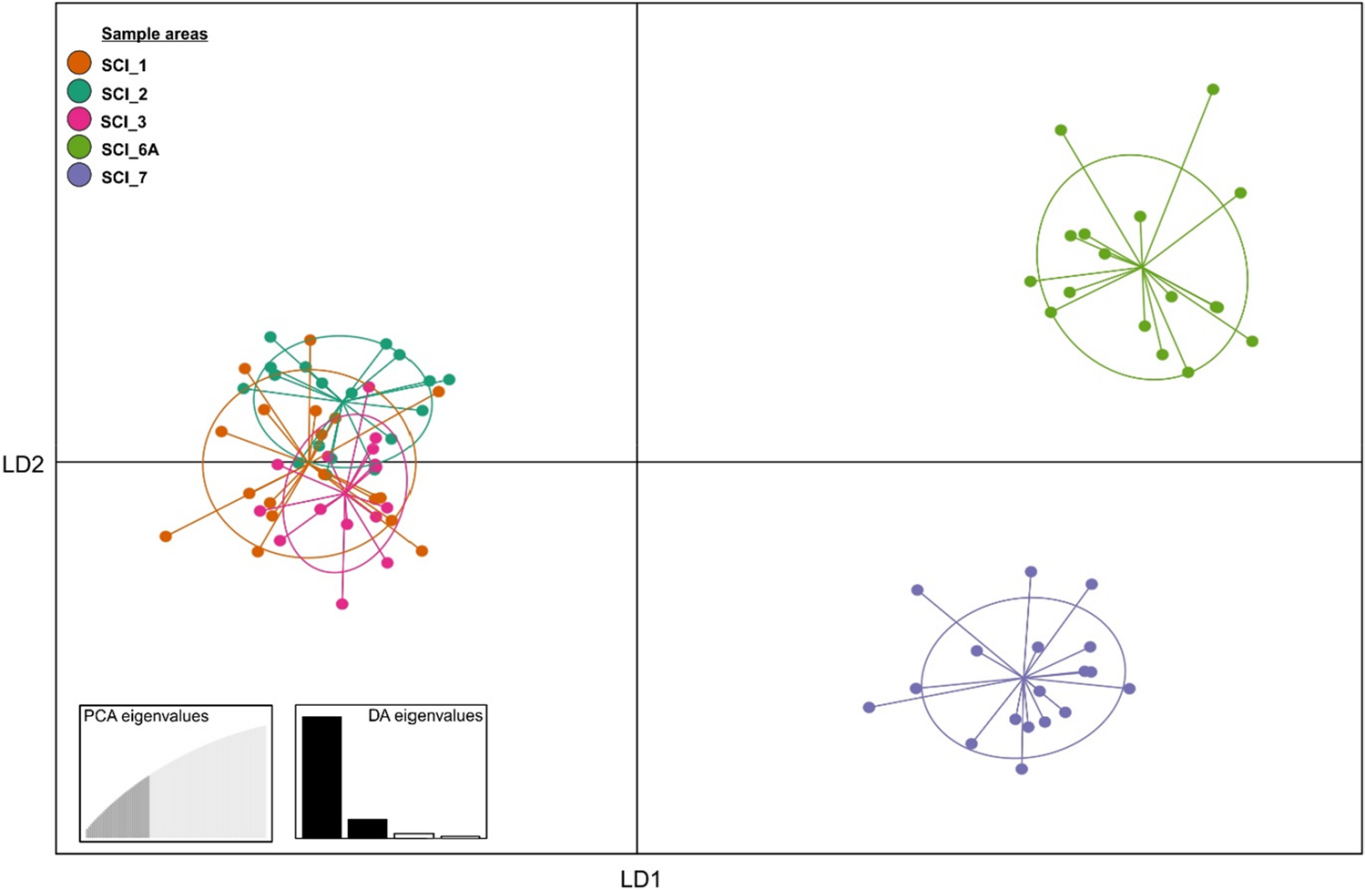
Scatterplot of the DAPC data showing the clustering of scampi individuals from their respective scampi fishery management areas (SCIs). SCIs are indicated by different colours, while dots represent individuals.

The Discriminant Analysis of Principal Components (DAPC) provided visualization of the three genetic clusters; SCI_E was separated from SCI_6A and SCI_7 along LD1 and the latter two SCIs were separated along LD2 (Fig. 2; this pattern of population structure was very similar to that shown in the PCA analysis—Figure S1). This clustering of individuals was also directly comparable with membership assignment probability, which can be a useful indicator of gene flow (Figure S2). The membership probabilities further supported the genetic similarity of individuals from SCI_1, SCI_2 and SCI_3. In contrast, there were high membership probabilities for individuals within SCI_6A and SCI_7, of which 94% from both SCIs were correctly assigned.

### Spatial structure

#### Migration rates

**Figure 3:**
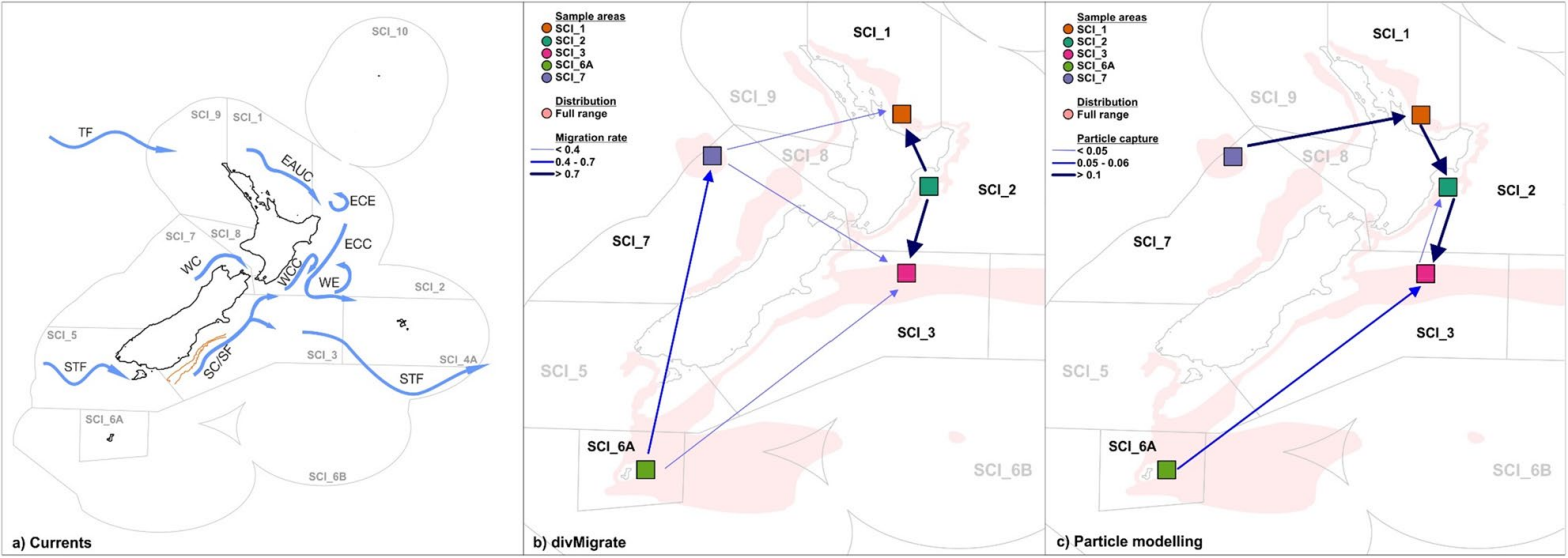
(**a**) Currents surrounding New Zealand that are likely to have influenced scampi migration rates between scampi stocks. (**b**) Migration rates estimated from genetic data using divMigrate and compared with (**c**) particle migration between adjacent scampi stocks from hydrodynamic modelling. (**a**) The direction of two of New Zealand’s major ocean fronts are indicated on the map, the Tasman Front (TF) and the Sub-Tropical Front (STF). Other major currents include the East Auckland Current (EAUC), the Southland Current/Front (SC/SF), the Wairarapa Coastal Current (WCC), the East Cape Current (ECC) and Westland Current (WC). Semi-permanent eddies embedded in the flows are the Wairarapa Eddy (WE) and East Cape Eddy (ECE). The Otago shelf edge is outlined in orange. The ocean current information was adapted from Chiswell and Booth^34^. (**b**) divMigrate relative migration rates between adjacent populations indicating higher rates of migration among SCI_1, SCI_2 and SCI_3, and between SCI_6A and SCI_7 (Table S8). The arrow direction points to which population is more likely to act as a sink among the adjacent SCIs. (**c**) The arrow direction indicates the resulting direction of particle movements from hydrodynamic particle modelling (Table S9). Indicated on the maps (**b**) and (**c**) are the sampling areas within each SCI (coloured square; Table S1), and the approximate full scampi range and fishery management areas^28^.The figure's main elements, i.e., New Zealand and sample areas, were generated with ggplot2 v3.3.3^114^, mapdata v2.3.0^115^ and mapproj v1.2.7^116^. Other aesthetic elements were added using Corel Draw Essentials X5 (v15.2.0.686).

The divMigrate results provide estimates of the relative migration rate, and where the direction in which the relative migration rate is larger indicates which SCI is likely to behave as a sink and the reverse for the source (Table S8; Figure S3). SCI_6A was found to behave as a source, whereas all other SCIs act as sinks with SCI_3 being the main sink (Fig. 3b; Table S8). The highest levels of gene flow were observed among SCI_E populations, and between SCI_6A and SCI_7. Importantly, the predominant directions of gene flow were from west to east, that is, from SCI_6A and SCI_7 towards SCI_E, and from SCI_1 to SCI_2 to SCI_3. However, there was no statistical significance to the asymmetric migration rates detected.

#### Particle modelling

**Figure 4:**
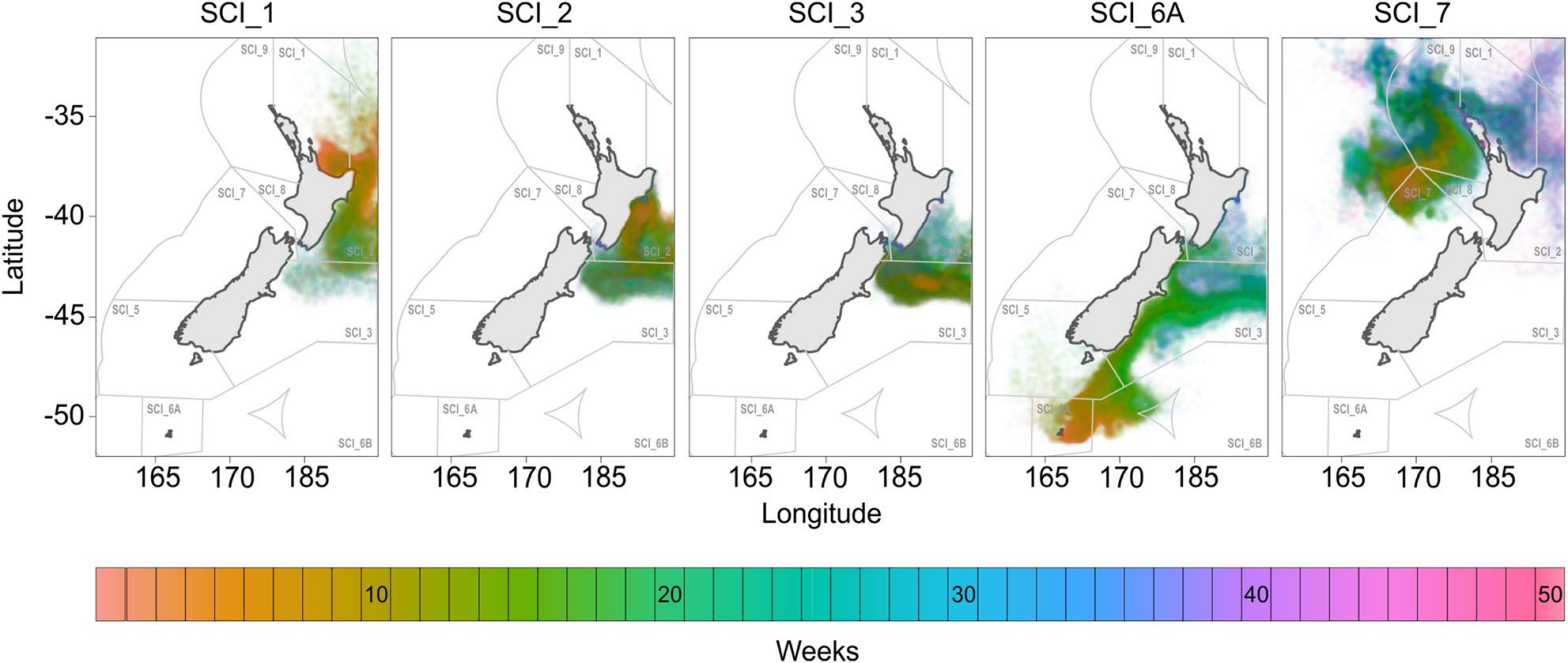
Modelled positions of particles in New Zealand waters recorded weekly up to approximately one year after being released from the five scampi fishery management areas (SCI_1, SCI_2, SCI_3, SCI_6A and SCI_7). Plotted is a random selection of 20,000 particles that were released divided equally per SCI, and their movement captured. The figure's main elements, i.e., New Zealand and position of particles, were generated with ggplot2 v3.3.3^114^ and rnaturalearth v0.1.0^117^. Other aesthetic elements were added using Corel Draw Essentials X5 (v15.2.0.686).

**Figure 5:**
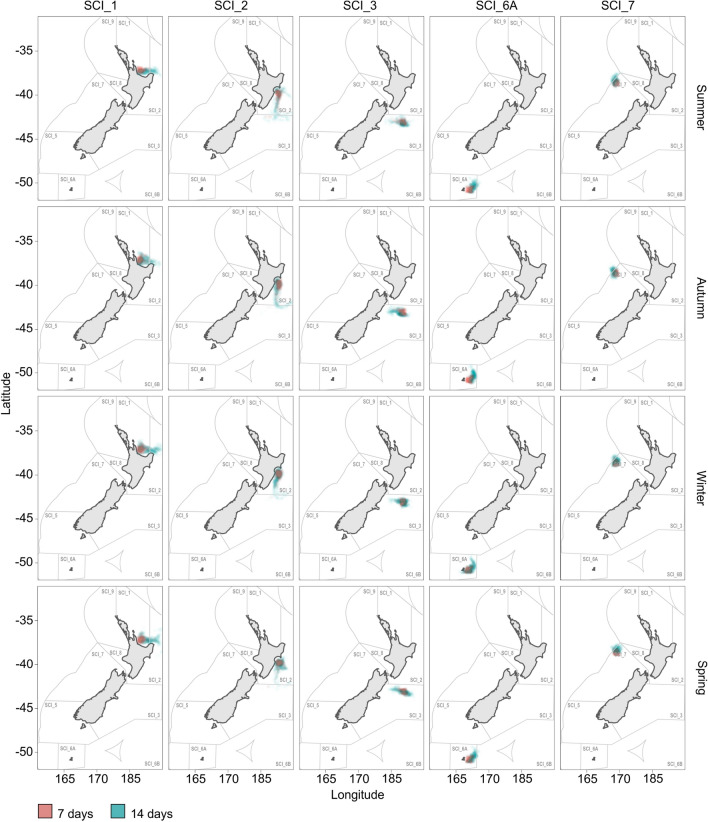
Modelled positions of particles in New Zealand waters after being released from the five scampi fishery management areas (SCI_1, SCI_2, SCI_3, SCI_6A and SCI_7). The particles positions are shown at seven (pink) and 14 (blue) days. Plotted is a random selection of 20,000 particles that were released divided equally per SCI, and their movement captured by season of release. The figure's main elements, i.e., New Zealand and position of particles, were generated with ggplot2 v3.3.3^114^ and rnaturalearth v0.1.0^117^. Other aesthetic elements were added using Corel Draw Essentials X5 (v15.2.0.686).

Particle modelling using the sophisticated Moana Backbone hydrodynamic model^45^ indicated a predominant directional flow of particles between locations following major current systems (Fig. 3a), from west to east (Figs. 4 and S4–S7). Particles released from SCI_1 reached SCI_2 (n = 6,701 particles captured and ${\overline{\text{x}}}$ = 13.2 weeks) and SCI_3 (n = 966 and ${\overline{\text{x}}}$ = 19.3), but in contrast, particles released from either SCI_2 or SCI_3 were very unlikely to reach SCI_1 (released from SCI_2: n = 4 and ${\overline{\text{x}}}$ = 27.8; SCI_3: n = 4 and ${\overline{\text{x}}}$ = 53.5) (Fig. 4; Tables S9 and S10). There was substantial movement of particles in both directions between SCI_2 and SCI_3 (release from SCI_2 and captured at SCI_3: n = 4,937 and ${\overline{\text{x}}}$ = 11.7; SCI_3 to SCI_2: n = 1,786 and x̅ 35.8) (Figs. 3c, 4), but more particles were captured when released from SCI_2 and captured at SCI_3. The Sub-Tropical Front (STF) extending eastward from the central South Island of New Zealand acts as a barrier preventing any particles moving from SCI_1, SCI_2 or SCI_3 to SCI_6A (Figs. 3a, 4). The STF also creates a barrier preventing particles from SCI_6A moving into SCI_7. The Tasman Front (TF) facilitates particle movement from SCI_7 around the top of the North Island to SCI_1 (n = 5,050 and ${\overline{\text{x}}}$ = 50.2). The majority of particles released from SCI_6A were carried by the currents to the east, but left the model domain, with about 10% captured within the sample areas in the eastern SCIs (SCI_1: n = 4 and ${\overline{\text{x}}}$ = 134; SCI_2: n = 1,131 and ${\overline{\text{x}}}$ = 37.5; SCI_3: n = 2,056 and ${\overline{\text{x}}}$ = 27.9). The number of particles reaching the sampling area within a SCI co-varied with the mean time period for particle arrival, i.e., where there were a greater number of arrivals, there was a shorter mean transit time. In all cases, except possibly for particle movements from SCI_2 to SCI_3, the shortest modelled transit time between sampled areas within SCIs was in excess of the PLD of scampi independent of season, although seasonal differences were identified with particles travelling different direct distances (i.e., straight-line distances measured between recorded points) in different seasons (i.e., > 11 days; Fig. 5; Table 4 and S9). The transit time is consistent with the estimates of particle migration representing patterns of averaged multi-generational larval dispersal, as could be expected via stepping stone processes whereby multiple successive generations occurring at intermediate locations are needed to transport new genetic material to more widely dispersed locations.

**Table 4:** Average distance simulated particles travelled in km over a 14 day period (pelagic larval phase is typically < 11 days) when released from the sampling locations during the different seasons. The distance is calculated by using direct distance between recorded locations.

Released from	Summer (km)	Autumn﻿ (km)	Winter﻿ (km)	Spring﻿ (km)
SCI_1	125	102	147	158
SCI_2	158	140	114	108
SCI_3	73	87	61	72
SCI_6A	88	92	66	73
SCI_7	63	55	52	63

## Discussion

Here we present one of the few cases that have integrated population genomics and particle modelling to deliver useful spatial and temporal insights into population connectivity for a deep sea species across a very large distribution range. This complementary approach illustrates how oceanographic drivers have the potential to influence genetic exchange among populations, including those separated by long distances. Many particle modelling studies have focused on the larval dispersal of shallow-water, coastal marine invertebrates (e.g., mussels^46,47^; scallops^35,48,49^; and lobsters^36,50^), which are typically influenced by more physically complex coastline and associated hydrodynamic processes compared with generally less complex bathymetry of deep water on continental shelf margins. In contrast, in deep sea species, which frequently have enormous distribution ranges, larval dispersal relies on broad scale hydrographic processes. Although more limited in the literature in comparison with coastal studies, particle modelling has proved to be particularly effective in the investigation of deep sea species population connectivity when combined with population genetics (e.g., deep sea corals^20,21^, sponges^51^ and mussels^25^), and as undertaken in this current study.

In this study, combining genomic data and physical modelling of particles shows that New Zealand scampi in SCI_1, SCI_2 and SCI_3 (all in close geographical proximity) consistently formed a single, relatively homogenous genetic cluster (SCI_E), distinct from both SCI_6A and SCI_7. This combined genetics/hydrodynamics approach identified patterns of gene flow that suggest a likely mechanism for persistence of these local populations. Specifically, modelling and genetic evidence indicates directional migration among stocks, with SCI_6A identified as a source population and SCI_3 identified as the main sink, providing important information to inform fisheries management.

### Population connectivity

We found significant genetic differentiation between the SCIs which fall into three clusters (SCI_E—east, SCI_6A—south and SCI_7—west; Tables 2, 3; Fig. 2). The genetic differentiation among SCIs was more subtle than might be expected for this species given their extensive spatial distribution and constrained capacity for dispersal due to their abbreviated pelagic larval phase, restricted post-larval benthic dispersal and low fecundity^26,29,31,33,52,53^. Significant genetic differences among eastern SCIs (SCI_1, SCI_2 & SCI_3) were found when utilizing a single mitochondrial marker^54^, but this differentiation was not apparent in this current study. The difference could potentially be due to the lower effective population size in mitochondrial DNA (haploid, and female inherited) versus nuclear DNA (diploid, and inherited from males and females), or due to the genome-wide analyses providing a more accurate evaluation of the stock structure, compared to the stochastically-variable results from a single mitochondrial gene^4,55^. It is also feasible that the difference is due to lower dispersal by female scampi, but this is unlikely, as there is no evidence to suggest that male scampi move significantly greater distances as adults^33^. Furthermore, for marine species which have large effective population sizes and weak population structure, differentiation due to genetic drift is slow to accumulate and can be offset by relatively small amounts of migration^56^.

It can be deduced that scampi habitat is present and occupied within the intervening seabed between our sampling locations which facilitates gene flow through the population by stepping through the intermediate populations where larval migration permits. This is consistent with frequent incidental catches of scampi in deep sea trawls for other species in areas not commercially fished for scampi (pers. comm., Jack Fenaughty, Silvifish Resources Ltd). A similar deduction of widespread suitable habitat was made for the Caribbean king crab, despite residing in shallow waters, its biological characteristics are similar to scampi in regards to having a low dispersal potential, no known long distance migrations and a large geographic range^39^. The study determined Caribbean king crab population connectivity using an integrative approach, such as in this study, and concluded that intermediate habitats must exist to maintain genetic exchange, allowing the local populations to remain connected through multiple generations, and that larval dispersal is influenced by oceanographic barriers^39^. The same conclusions can be made regarding the population connectivity of scampi. The degree of connectivity and the direction of gene flow among SCIs, as estimated by both the genetic and particle modelling methods, are greatly influenced by the predominant ocean current regime around New Zealand that influences larval dispersal. The parameters set for the particle modelling effectively estimated an averaged multi-generational larval dispersal that would characterise stepping stone processes and in this regard is coherent with the independent assessment derived from genetic estimates.

Both genomic and hydrodynamic modelling data indicate a predominantly west to east directional connectivity in the scampi population. The direction of connectivity suggests that some stocks act as net sources or sinks. Both datasets strongly indicate that SCI_6A acts as a net exporter of larvae (source) and that SCI_3 acts as a net recipient of larvae (sink) with the extent of their respective larval supply likely to be shaped by the hydrodynamic regime, as well as the extent of intermediate scampi population involved in a stepping stone transfer process (Figs. 3, 4). Furthermore, given the observed patterns of particle dispersal, SCI_3 would likely act as a source of migrants for the Chatham Islands management area to the east (SCI_4, unsampled in the present study). The advantage of being a sink stock (e.g., SCI_3) is that the increased genetic diversity potentially provides greater resilience, adaptability, fitness and evolutionary flexibility, which would promote long term population survival^57,58^. Sink stocks typically rely on recruitment from source stocks (e.g., SCI_6A) that have sufficiently large effective population sizes and self-recruitment to also maintain themselves. Overexploitation of source stocks can have implications for the viability of the wider population, as it can decrease not just local recruitment but the recruitment, productivity and persistence of associated sink stocks^59,60^.

As has been noted in this study and in other deep sea studies (e.g., coral^20,21^ and sponge^51^), hydrodynamic processes play an important role in population connectivity and particular features (e.g., eddies) shape the connectivity by directing larvae dispersal and/or increasing or decreasing the dispersal range. The STF appears to permit only a unidirectional gene flow from SCI_6A to SCI_3, while greatly restricting particle movement from SCI_3 to SCI_6A. These results are concordant with previous studies in the species using a single COI marker that suggested this marked genetic differentiation between scampi from the Auckland Islands (SCI_6A) and those east of the main islands of New Zealand is likely due to the STF^30,54^. Our results also suggest that the TF also likely plays a role in facilitating gene flow from the west to the east (Fig. 3a). These large scale ocean currents tend toward barotropic flow which would serve to transport larval particles in a coherent manner regardless of their vertical position in the water column^45^.

The genetic measure of connectivity (divMigrate) estimated asymmetric relative rates of migration based on patterns of allelic differentiation among SCIs. The direction of migration is determined by measuring the extent of shared alleles and can become less definitive when the majority of alleles are shared among populations. Hydrodynamic modelling of the movements of particles (i.e., proxy larvae) provides an independent measure of the likely extent and direction of connectivity among locations within the population range. The particle modelling indicated high levels of exchange between SCI_2 and SCI_3, although favouring particle movement from SCI_2 to SCI_3 versus SCI_3 to SCI_2, which corresponds with the measures provided by the genetic migration method (Fig. 3). The exchange from SCI_3 to SCI_2 is likely facilitated by both the strong circulation of the Wairarapa Eddy (WE) and the East Cape Current (ECE) (Figs. 3a, 4).The WE and ECE are two eddy systems that cover a large geographical area, persisting throughout the water column, and can retain weak swimming larvae long enough for them to reach settlement stage^34,61,62^. These characteristics also contribute to the sink status of SCI_3^34,62–64^. Particle modelling further indicates that the direction of movement favours SCI_1 to SCI_2 versus SCI_2 to SCI_1, which is at odds with the genetic migration results (Fig. 3b). Although, when taking into account that SCI_2 receives larvae from SCI_1 and SCI_3 we believe that it is evident that the shared alleles and low level of genetic differentiation among these populations creates a bias in the genetic migration estimates, indicating the direction is from SCI_2 to SC_1 and SCI_3. Therefore, the particle modelling also allows for a refined understanding of the process behind the observed genetic patterns^20^ and can correct wrong assignments of source populations^25^ when molecular markers are not sufficient to resolve population structure.

The patterns of likely gene flow inferred by the particle modelling largely reflect the known distribution range of the species (Fig. 1), although it is perhaps surprising that there is such little genetic divergence between SCI_6A and SCI_7, given the apparent geographic isolation of the sampled region in SCI_7. This suggests that genetic drift among SCIs may be limited due to the large effective population sizes^54^, or more likely that the two areas are linked through a contiguous distribution of this species that permits stepping stone gene connectivity when supported by hydrodynamic dispersal of larvae (Fig. 4; Table S9). There is evidence that such contiguous populations of scampi exist along the west coast of the South Island, as scampi are often captured incidentally in fish trawls between SCI_6A and SCI_7 (pers. comm. Jack Fenaughty, Silvifish Resources Ltd; Fig. 1). In general, the presence of a contiguous scampi distribution may be restricted by suitable habitat area (e.g., narrow stretch of the Otago Shelf; Fig. 3a) and/or lack thereof resulting in dispersal being less than that predicted from particle modelling, and thereby reducing gene flow. The reduced gene flow is likely to contribute to the degrees of significant genetic differentiation seen among SCIs^25^.

### Fisheries implications

Habitat suitability can vary due to spatial/environmental heterogeneity, with some habitats being more suitable for particular species than others^65^ (e.g., food availability, refuge quality, predator/competitor density^66^); allowing a species to thrive rather than survive. These factors help to drive source-sink dynamics, but habitat suitability can change via natural or anthropogenic causes^67,68^. These changes can be to the detriment (trawling resulting in habitat fragmentation) or advantage (fisheries removing predators promoting prey species survival) of the population, as changing the habitat size and quality directly affects population stability and persistence^67,69^. In regards to species that have a short dispersal period, they would settle ‘near’ to their release, but this limits them to the quality of the habitat in the immediate area and typically strengthens source-sink dynamics^65,69^.

Source-sink dynamics are key ecological features with important implications for fisheries stock recruitment/management as connectivity influences both sustainability and yield^6,27,48,68^. Furthermore, dismissing or failing to identify source-sink dynamics can lead to flawed decision making that can ultimately affect species abundance and distribution^70^, as identifying connectivity patterns will help determine which local stocks rely on larval retention and/or immigration. This connectivity information allows for each stock’s contribution to the overall population to be characterised, as well as the potential for the population to persist, and can help with potential decision making regarding setting/reassessing stock quota, restocking or replenishment of depleted populations^27,41,48,71^.

In this study, scampi movement and exchange patterns were used to indicate source-sink dynamics. The potential for fishing to impact on the population connectivity inferred here should be considered in the management of this fishery, along with the variable population vulnerability (i.e., source or sink). When allocating catch quota to SCIs, it is also important to recognise the distinct genetic sources and safeguard them. Source-sink research on scallop populations have argued that when tailoring catch limits by considering contribution to recruitment, there is an added benefit that higher yields could be made available for fishers at a lower risk of over exploitation^35^. Work on other lobster species has identified that larval supply is dependent on surrounding areas and thus fishers depend on the protection of these sources to protect the stocks^72^. Furthermore, these results suggested that moderate changes to lobster management areas needed to be made whereby sinks reliant on the same sources are grouped^72^. Finfish studies have recommended similar management incorporating source-sink dynamics and have shown that a decrease in the exploitation in source populations (without reallocating quota) has favourable outcomes on adult survival and recruitment in sink populations^73^.

The exploitation of New Zealand scampi sources should be considered in the context of their dependence on high internal recruitment and the importance of providing immigrants that contribute to sustaining the wider population. For example, the two SCIs of greatest fisheries importance, SCI_6A and SCI_3, have similar commercial catch allocations (total allowable commercial catch—TACC). SCI_6A is 306 t and SCI_3 is 408 t; these two SCIs making up 54% of all TACC allowed for scampi, but have contrasting source-sink features^26^. SCI_6A appears to rely on local recruitment to a much greater extent, and behaves as a source population based on inferences of directional gene flow. Furthermore, it has been estimated that individuals from SCI_6A have higher levels of emergence, as they appear to spend less time in their burrows, increasing their catchability by benthic trawl nets^33,74^. Both these features potentially make this stock more vulnerable to over-exploitation and more important to ensure its persistence. In contrast, SCI_3, being the main sink, is more dependent on immigration from other stocks. Therefore, differential management should be considered for sources and sinks to avoid major consequences to stocks, as strong sources create strong sinks^68,69^.

Ocean circulation strongly influences genetic connectivity and a shift in current direction and intensity can ultimately change stock connectivity. There is evidence for changes in velocity of some ocean currents due to anthropogenic climate change^75–78^. These changes can add a layer of difficulty to fisheries management over time, and monitoring will be needed to ensure management accommodates potential alterations in larval dispersal. Modelling has predicted the complexity of ocean circulation changes in the Tasman Sea and it is projected that, by the end of the century, the southern branch of the East Australian Current (EAC) will intensify and that the TF will weaken^78^. This change may reduce the EAC flow, potentially reducing long-term gene flow to the eastern SCIs. Global climate change has a very real potential of disrupting larval dispersal pathways and impeding population connectivity maintained through ocean currents^6^. This is not only limited to the changing of ocean currents, but the rise in sea temperature is also of concern among other changes (e.g., ocean acidification). Larval duration may be reduced, as an increase in larval development rate due to an increase in water temperature, may further alter dispersal capabilities and population connectivity^6,79–83^.

This research has identified areas where more information is required to better understand the scampi population interactions in relation to fishery implications. Greater confidence in the currently inferred patterns could be achieved by genetically analysing a greater number of individuals at each site, improving the accuracy and statistical power of the estimated genetic parameters. In addition, the degree and extent of stepping stone gene flow could be tested through inclusion of additional, intermediate sampling locations which would provide better inferences across the species’ range. These measures will help add detail to the broad population structure that can aid in fisheries conservation and management decisions.

## Conclusion

This study on the population structure of New Zealand scampi successfully used both genetic data and hydrodynamic particle modelling to independently quantify principal patterns of dispersal and connectivity. The genetic data revealed significant stock differentiation among SCI_E, SCI_6A and SCI_7. Both genetics and hydrodynamic modelling methods reveal a strong predominant pattern of directional dispersal around New Zealand, following the major currents, and showing an overall eastward dispersal of larvae. Hydrodynamic modelling identified SCI_6A as a potential source stock, and SCI_3 was identified as the main sink. Genomic data was less definitive as to the directions of dispersal, but still supported the major patterns identified by the modelling. This combined approach is transferable to other exploited and ecologically important species that rely heavily on hydrodynamic drivers for dispersal and genetic connectivity, and the knowledge gained can assist in better understanding of population connectivity to make more appropriate and effective conservation and fisheries management decisions e.g.,^21,39,42,43,50,51,84^.

## Methods

A detailed version of the methods has been included in the supplementary material to ensure reproducibility, which includes the specifics of which functions were used from the respective packages and any parameter changes from the default values.

### Sample collection

Scampi were collected from five scampi SCIs within New Zealand’s exclusive economic zone, namely from the east: SCI_1, SCI_2 and SCI_3, south: SCI_6A and west: SCI_7, using bottom trawling methods between April 2017 and April 2018 (Fig. 1; Table S1). The scampi were provided by Waikawa Fishing Company Ltd (Blenheim, New Zealand): 20–30 scampi were randomly selected per trawl once landed. They were then rapidly cooled in salt ice slurry until becoming torpid and subsequently preserved in 95% ethanol.

A special permit (#549) for scampi collection was provided by New Zealand’s Ministry for Primary Industries. The specimens for this study were collected in accordance with approvals under New Zealand’s Animal Welfare Act 1991 approved by the Animal Ethics Committee of the Nelson—Marlborough Institute of Technology (AEC2014-CAW-02). Scampi are taonga (treasured) species for Māori and as such this research is part of a larger project that has a core focus on transdisciplinary research engaging Mātauranga Māori and western science^85^.

### GBS sample preparation

DNA extraction was undertaken on scampi tail muscle tissue, using the Gentra Puregene Tissue Kit (Qiagen, Hilden, Germany) following the manufacturer’s instructions. Based on quality (0.8% agarose gel visual) and quantity (Qubit dsDNA HS Assay Kit—Invitrogen, Thermo Fisher Scientific Inc., Waltham, MA, USA) assessments from a total of 171 individuals, 91 individuals were selected for GBS. Of these, 18 individual scampi were selected from each of SCI_1, SCI_2 and SCI_3, 17 individuals from SCI_6A, and 20 individuals from SCI_7. Additional DNA extractions from two individual scampi from SCI_7 were used as quality control (QC) replicates. Further preparation and sequencing of the samples for GBS was conducted by AgResearch Ltd (Dunedin, New Zealand). Sequencing was undertaken on an Illumina HiSeq 2500 (Illumina, San Diego, CA, USA) 1 × 101 cycle utilizing v4 chemistry.

### Quality control and de novo genotyping

FASTQC v0.11.7^86^ was used to analyse the quality of the raw sequencing data. PROCESS RADTAGS, a module in STACKS v2.4^87^, was used to demultiplex the data and undertake quality control by removing reads with (1) low quality (< 90% probability of being correct), (2) no barcodes or restriction enzyme cut-sites, (3) < 75 bp (reads truncated if > 75 bp), (4) uncalled base and (5) low quality scores. Reads were kept only when a single mismatch occurred in barcodes and restriction enzyme sites. The reads retained were passed to further STACKS modules in succession for de novo genotyping. To ensure only recurring loci were included in subsequent analyses, the loci that were present in at least 80% of individuals in each SCI and present in all SCIs were selected. The catalogue and genotyping of samples were undertaken on the same run.

### Data filtering

#### Quality filtering

The STACKS haplotypic dataset was used for data filtering and analyses. The QC replicates were assessed and the replicate that was of lower quality was excluded from further analyses. Individual and SNP statistics were produced with VCFTOOLS v0.1.14^88^ using the STACKS SNP dataset to assess dataset quality. The data were examined for missing data for both individuals and SNP sites, allele counts and frequencies, and sequencing depth per SNP site. The loci underwent further QC in R v4.1.0^89^. The SD and mean locus sequencing depth were calculated, and loci that were two SDs above the mean were excluded from the dataset to remove potential repetitive sequence regions. Remaining loci that had more than five SNPs per locus were also excluded as a precaution against paralogs. These excluded loci were filtered from the STACKS haplotype dataset in order to remove potential false genotypes due to DNA sequencing errors^90,91^. Please see supplementary methods for details regarding Hardy–Weinberg equilibrium departure filtering and outlier filtering as to why it was not incorporated.

### Patterns of genetic diversity and differentiation

#### Genetic diversity statistics

Summary statistics were calculated per SCI per locus using HIERFSTAT v0.04-22^92^. The observed (H_o_) and expected (H_e_; gene diversity) heterozygosity for each SCI was calculated, as well as the inbreeding coefficient (F_IS_)^93^. Allelic richness (A_r_) was also measured, which uses a rarefied measure of the number of alleles at each locus and in each SCI. The mean nucleotide diversity (π) was estimated per SCI using the POPULATIONS module (STACKS) output data for only those loci that passed QC (i.e., only variable loci). The initial statistical results indicated a clustering of SCI_1, SCI_2, and SCI_3 (SCI_E) and so this strata was included in the statistical analyses.

#### Genetic differentiation and population structure

Differentiation indices and multivariate statistical approaches were used to investigate genetic differentiation and population structure. Firstly, pairwise and overall F_ST_^94^ values amongst SCIs were calculated (STRATAG v2.0.2^95^; *p* values were adjusted with a Bonferroni correction—STATS v3.6.1^89^) by running a total of 10,000 permutations. An analysis of molecular variance (AMOVA; POPPR v2.8.3^96,97^) was undertaken to investigate the portion of variance within and among SCIs to further explore population structure. Significance was assessed using a randomization test based on 100,000 repetitions.

The population structure was analysed by applying the multivariate statistical method of a DAPC, which identifies and describes clusters of genetically related individuals (ADEGENET v2.1.1^98,99^). This method also enabled the assignment of individuals to clusters and the contribution of individual alleles to population structuring, thereby characterizing population subdivision. Individual assignment utilized the true sampling locations as clusters (SCI priors given). A PCA was also performed to ensure the de novo DAPC population structure reflected an accurate representation of the overall population structure, despite migration rates. It has been found that when migration rates are high and groups are described de novo using a DAPC there may be substantial inaccuracy^100^.

### Spatial structure

#### Migration rates

DIVERSITY’s v1.9.90^14,101^ divMigrate was used to test and understand the influence that ocean currents have had on suspected directional asymmetric migration pattern among populations, and ultimately direction of gene flow. Patterns of allelic differentiation were used to estimate asymmetric relative rates of migration. G_ST_^102^ was the statistical parameter selected to estimate the relative migration between populations. The mean relative migration rates and 95% confidence intervals were calculated using 10,000 bootstrap iterations.

#### Particle modelling

Modelling of the movement of particles (i.e., proxy larvae) was undertaken with the open source particle tracking framework OPENDRIFT v1.0^103^ driven by the most sophisticated 3-dimensional hydrodynamic model for New Zealand’s ocean region (Moana Project New Zealand backbone v1.9^45^). This model is based on, and calibrated against, more than 25 years of detailed hydrodynamic hindcast data. The model uses high resolution bathymetry available for the shelf waters for the New Zealand region (i.e., General Bathymetric Chart of the Oceans (GEBCO) in combination with higher resolution locally-sourced data) and resolves 50 vertical sigma layers in the water column. The particle tracking hydrodynamic model was used to simulate the dispersal of scampi larvae during their pelagic phase from the sampling locations and the potential inter-generational connectivity trends among the scampi sampling locations, as a proxy for genetic exchange, and assumes the availability of viable intermediate scampi habitat where arriving genetic material can establish and reproduce to further disperse the genetic material. Thus, the particle dispersal model was intentionally run over an extended period (10 years) to account for inter-annual variability in hydrodynamic processes so as to capture dispersal patterns in an analogous fashion to that of genetic connectivity estimates, providing an overall averaged characteristics of circulation^20^. The model domain covers New Zealand’s Exclusive Economic Zone (model domain coordinates: 161.03°, − 31.03°; 184.97°, − 31.03°; 161.03°, − 51.98°; 184.97°, − 51.98°) and places the open boundaries far enough to fully encompass the TF to the north and the STF to the south whilst also maintaining practicalities of the computational cost for the provision of high resolution models of this scale. Due to the poor swimming abilities of scampi throughout their larval development^29,104^ they were treated as passive particles in the model, moved by the 3-dimensional hydrodynamic forces generated from the model and a diffusion coefficient.

We selected particle tracking parameters based on previous hydrodynamic modelling of invertebrate larval dispersal in New Zealand^46,105,106^. One hundred representative particles were released on the surface from evenly spaced locations within each sampled area (encapsulating the trawl area; Fig. 1) on each release step (every 6 h between 1 January 2008 and 31 December 2008; n = 146,100), and their movements tracked to estimate the mean direction and duration of particle movement between SCIs. Particles were released over a year as scampi are presumed to release eggs year-round, as berried females are captured throughout the year. Particles were tracked using the model until they met one of three conditions (1) the particles left the model domain, (2) they reached one of the other sample areas, or (3) until 31 December 2017, i.e., 10 year period. The model operated with an internal timestep of 15 min and particle locations were recorded every seven days. A diffusion coefficient of 0.18 m^2^ s^−1^ was included to account for sub grid scale diffusion based on the equations of Okubo and Ebbesmeyer^107^. The raw particle modelling data was extracted using NCDF4 v1.17^108^. The data was used in several ways, (1) to extract the data pertaining to that of the pelagic larval phase per season (December to February—summer; March to May—Autumn; June to August—Winter; September to November—Spring), (2) to track the movement of the particles which could then provide details of the approximate number of generations needed for connectivity among dispersed locations, and (3) enumerating connectivity by calculating the total number of particles arriving at each SCI sample area from each source sample area over the modelling period (i.e., source-sink dynamics). Our use of hydrodynamic models for simulating larval dispersal draws on well-established research for a wide range of marine species e.g.,^109–112^. However, our approach to the application of the modelling differs in that we are interested in multigenerational connectivity which is consistent with the biological process for migration of genes within a wide ranging population, and it ensured that inter-annual variability in hydrodynamic processes were incorporated into the modelled outcomes, whilst also ensuring there was enough time for particle dispersal to assess possible long distance multigenerational dispersal.

## Supplementary Information

Supplementary Information 1.

Supplementary Information 2.

## Data Availability

All the data used in this study is openly available. The raw GBS data (fastq files) has been uploaded to the NCBI Sequence Read Archive (http://www.ncbi.nlm.nih.gov/bioproject/760674) with the associated metadata. This data is also available on figshare along with the haplotypic VCF file produced by STACKS and the particle modelling data (NETCDF files) produced by OPENDRIFT (https://doi.org/10.17608/k6.auckland.13152932). This data has also been captured on GEOME (https://geome-db.org/workbench/project-overview/?projectId=425).

## Code availability

The code for the data analyses is available on GitHub (https://github.com/AvdReis/NZ_scampi_GBS).
