# Supplementary material for: Genetic and particle modelling approaches to assessing population connectivity in a deep sea lobster
Source: Sci Rep. 2022 Oct 6;12:16783. doi: 10.1038/s41598-022-19790-5 (PMC9537507; doi:10.1038/s41598-022-19790-5)
Supplement: Supplementary file 2 — Supplementary Information 2. [file 41598_2022_19790_MOESM2_ESM.docx]

**Genetic and particle modelling approaches to assessing population connectivity in a deep sea lobster**

Authors: Aimee L. van der Reis*^1 3^, Craig R. Norrie ^2^, Andrew G. Jeffs ^1^ ^3^, Shane D. Lavery ^1^ ^3^ and Emma L. Carroll ^3^

^1^ Institute of Marine Science, University of Auckland, New Zealand

^2^ School of Aquatic and Fisheries Sciences, University of Washington, United States

^3^ School of Biological Sciences, University of Auckland, New Zealand

# Methods (detailed version)

## **Sample** collection

Scampi were collected from five scampi FMAs (officially referred to as SCI – scampi fishery management area) within New Zealand’s exclusive economic zone, namely from the east: SCI_1, SCI_2 and SCI_3, south: SCI_6A and west: SCI_7, using bottom trawling methods between April 2017 and April 2018 (Figure 1; Table S1). The scampi were provided by Waikawa Fishing Company Ltd (Blenheim, New Zealand): 20 - 30 scampi were randomly selected per trawl once landed. They were then rapidly cooled in salt ice slurry until becoming torpid and subsequently preserved in 95% ethanol.

A special permit (#549) for scampi collection was provided by New Zealand’s Ministry for Primary Industries. The specimens for this study were collected in accordance with approvals under New Zealand’s Animal Welfare Act 1991 approved by the Animal Ethics Committee of the Nelson - Marlborough Institute of Technology (AEC2014‑CAW-02). Scampi are taonga (treasured) species for Māori and as such this research is part of a larger project that has a core focus on transdisciplinary research engaging Mātauranga Māori and western science^1^.

## GBS sample preparation

DNA extraction was undertaken on scampi tail muscle tissue, using the Gentra Puregene Tissue Kit (Qiagen, Hilden, Germany) following the manufacturer’s instructions. DNA quality was assessed by visualization of DNA in a 0.8% agarose gel stained with Gel Red (Biotium, Fremont, CA, USA), in a Gel DocTM XR+ (Bio-Rad Laboratories Inc., California, USA) and DNA quantity was estimated using Qubit dsDNA HS Assay Kit (Invitrogen, Thermo Fisher Scientific Inc., Waltham, MA, USA) following the manufacturer’s instructions. Based on these quality and quantity assessments from a total of 171 individuals, 91 individuals were selected for GBS. Of these, 18 individual scampi were selected from each of SCI_1, SCI_2 and SCI_3, 17 individuals from SCI_6A, and 20 individuals from SCI_7. Additional DNA extractions from two individual scampi from SCI_7 were used as quality control (QC) replicates.

Further preparation of the samples for GBS was conducted by AgResearch Ltd (Dunedin, New Zealand). Briefly, library preparation utilized restriction enzymes *SbfI* (rare cut-site) and *MspI* (common cut-site) for a double digest of each individual's DNA^2^. Adapters were then ligated allowing each individual’s DNA fragments to have a unique barcode. Barcoded samples were pooled into a single tube and purified (Qiagen’s QIAquick PCR Purification Kit). A PCR step was implemented to increase the copy number of DNA fragments. The PCR reaction was slightly modified from Elshire et al.^3^, but the primers and PCR protocol were the same as per the restriction fragment amplification section. Each PCR reaction contained 4 µl DNA, 25 µl Taq 2× Master Mix (New England Biolabs Inc., Ipswich, MA, USA), 1 µl of each primer (25 pM) and 19 µl H_2_O. The resulting PCR product underwent clean-up (QIAquick PCR Purification Kit Protocol). Size-appropriate fragments were selected, 150 - 500 base pairs (bp), using a Pippin Prep (Sage Science Inc., Beverly, MA, USA). Sequencing was undertaken on an Illumina HiSeq 2500 (Illumina, San Diego, CA, USA) 1 x 101 cycle utilizing v4 chemistry.

## Quality control and *de novo* genotyping

FASTQC v0.11.7^4^ (all software/R packages are denoted similarly) was used to analyse the quality of the raw sequencing data from the Illumina HiSeq 2500 run. PROCESS RADTAGS, a module in STACKS v2.4^5^, was used to demultiplex the data and undertake quality control by removing low quality reads (< 90% probability of being correct), as well as reads with no barcodes or restriction enzyme cut-sites. The reads were also removed if they were shorter than the minimum length of 75 bp or truncated if read length was greater than 75 bp. The clean, quality and rescue parameters were implemented, thus removing any read with an uncalled base, discarding reads with low quality scores and correcting barcodes and restriction enzyme sites only when a single mismatch occurred, respectively.

The reads that passed QC in PROCESS RADTAGS module were passed to further STACKS modules in succession for *de novo* genotyping, as per the STACKS manual, given the lack of a suitable, available reference genome. Only three of these STACKS modules had their default parameters or input file changed. In the USTACKS module the number of identical reads needed to qualify as a stack was increased (*m = 5;* all parameters are denoted similarly). In the TSV2BAM module individuals with low genotyping success (defined as individuals having less than 3,000 loci matched to the catalogue; Table S2) were identified and not considered further by subsequent modules (modification to default population input file). To ensure only recurring loci were included, the loci that were present in at least 80% of individuals in each SCI and present in all SCIs (POPULATIONS module; *r = 0.8*; *p = 5*) were selected. The catalogue and genotyping of samples were undertaken on the same run.

## Data filtering

### Quality filtering

The STACKS haplotypic dataset was used for data filtering and analyses. The QC replicates were assessed and the replicate that was of lower quality was excluded from further analyses. Individual and SNP statistics were produced with VCFTOOLS v0.1.14^6^ using the STACKS SNP dataset to assess dataset quality. The data were examined for missing data for both individuals and SNP sites, allele counts and frequencies, and sequencing depth per SNP site. The loci underwent further QC in R v4.1.0^7^. The standard deviation (SD) and mean locus sequencing depth were calculated, and loci that were two SDs above the mean were excluded from the dataset to remove potential repetitive sequence regions. Remaining loci that had more than five SNPs per locus were also excluded as a precaution against paralogs. These excluded loci were filtered from the STACKS haplotype dataset in order to remove potential false genotypes due to DNA sequencing errors^8,9^.

### Hardy-Weinberg equilibrium departure filtering

The assumptions of Hardy-Weinberg equilibrium (HWE) are rarely met in real biological populations^10^ and HWE departure filtering, and the varying filtering conditions, may have a dramatic effect on population genetic inferences^11^. HWE departure filtering often aids in removing loci with genotyping errors, however, the filtering process used in this study was stringent which removed concerns regarding genotyping errors, which was further satisfied by the high read coverage per loci^10,12^. In addition, while our sample size is suitable for the current study, filtering for HWE is not recommended when sample sizes are ‘small’ as the power to detect departures is lower^10,11^. Furthermore, current recommendations suggest no filtering is necessary for the detection of population structure in datasets and appropriate (non-bias) filtering only marginally increases the ability to detect population structure in datasets^11^. Therefore, the ability to estimate the general population divergence trends and avoid introducing biases into the dataset is achievable by refraining from using HWE departure filtering, as done in this study.

### Outlier filtering

The detection of outliers is a well-known method to assist in identifying loci that have potentially undergone adaptation and associated genes/genomic regions that are possibly under selection^13,14^. Four approaches were used to identify potential outliers following recommendations to utilize different approaches^15-17^.

BAYESCAN v2.1^18^ is based on the multinomial Dirichlet likelihood model^19^ which implements a Bayesian approach and using logistic regression decomposes the locus-population F_ST_ coefficients into a population-specific component and a locus-specific component. Default parameters were kept except for prior odds parameter was increased to 100 to increase the elimination of false positives. The resulting data was analysed in R and loci were determined to be under the influence of selection if they had a q-value < 0.1.

OUTFLANK v0.2^17^ is an R package that finds outliers through trimming the distribution (likelihood approach) of F_ST_ values to infer the distribution of F_ST_ for neutral markers. ***MakeDiploidFSTMat*** (all functions are denoted similarly) calculates the F_ST_ ^20^ values without sample size correction and then ***OutFLANK*** implements the trimmed likelihood approach, while the only change to the default parameters was that the q-value threshold was changed to 0.1 to allow for direct comparison with other methods used.

In LEA v2.2.0^21^ the sparse non-negative matrix factorization (sNMF)^22^ was used to estimate population structure by estimating individual admixture coefficients from a genotype matrix. ***snmf***, which is a function that is very close to Bayesian clustering programs and provides least-squares estimates of ancestry proportions, was used to estimate the number of ancestry populations (i.e., number of distinct genetic clusters). The cross-entropy criterion was calculated with values of K = 2-5 with each K value having 100 repetitions, the maximum number of iterations in the algorithm set to 500 and the regularization parameter (alpha) set to 200. Admixture coefficients were run for K = 2-5 using the smallest cross-entropy value from the 100 repetitions for each K value (Figure S8). A genome scan then identified adaptive alleles using population differentiation statistics^23^ which are based on a selected K value that best captures the main pattern of differentiation in the genotypic data (K = 2), and p-values are computed for each locus. Diagnostic markers were identified using ***snmf.pvalues*** (*genomic.control = TRUE*) using the output from ***snmf*** with K = 2. The p-values were transformed into q-values using the R package QVALUE v2.15.0^24^ and markers were detected using a q-value threshold of 0.1.

The DAPC’s principal components (ADEGENET) reflect between-population variability and can be used to identify alleles (thus loci) that are potential diagnostic markers. Alleles were retained that were greater than 0.009 loading for the first linear discriminant function (see var.contr - ***dapc*** object). The 0.009 threshold was visually determined, but outliers were then also compared to BAYESCAN, OUTFLANK and LEA results for reliability.

The consensus sequence for each outlier locus was run through the National Center for Biotechnology Information (NCBI) GenBank database^25^, using nucleotide BLAST (BLAST v2.6.0) megablast^26^. A locus was only determined to be under selection if all approaches identified it as an outlier. As only a single outlier was identified by all analyses providing very weak evidence for selection, no filtering took place and the outlier were retained in the dataset for further analysis.

## Patterns of genetic diversity and differentiation

The R package RADIATOR v1.1.2^27^ was used for the majority of file format conversions for the appropriate R packages and software used.

### Genetic diversity statistics

Summary statistics were calculated per SCI per locus using HIERFSTAT v0.04‑22^28^. Using the function ***basic.stats*** the observed (H_o_) and expected (H_e_; gene diversity) heterozygosity for each SCI was calculated, as well as the inbreeding coefficient (F_IS_)^29^. Allelic richness (A_r_; ***allelic.richness***) was measured, which uses a rarefied measure of the number of alleles at each locus and in each SCI. The mean nucleotide diversity (π) was estimated per SCI using the POPULATIONS module (STACKS) output data for only those loci that passed QC (i.e., only variable loci). The initial statistical results indicated a clustering of SCI_1, SCI_2, and SCI_3 (SCIs that are predominantly east of the main islands of New Zealand; collectively named ‘SCI_E’) and so this strata was included in the statistical analyses.

### Genetic differentiation and population structure

Differentiation indices and multivariate statistical approaches were used to investigate genetic differentiation and population structure. Firstly, pairwise and overall F_ST_^30^ values amongst SCIs were calculated (***popStructTest***; STRATAG v2.0.2^31^) by running a total of 10,000 permutations. The pairwise p-values were adjusted with a Bonferroni correction (***p.adjust***; STATS v3.6.1^7^)

An analysis of molecular variance (AMOVA; ***poppr.amova***; POPPR v2.8.3^32,33^) was undertaken to investigate the portion of variance within and among SCIs to further explore population structure. A pairwise distance matrix between SCIs was calculated (allele counts were converted to frequencies before the distance was calculated) and transformed with the positive eigenvalues of the Euclidean representation (i.e., no biological assumptions). The changes from default settings for the function were *method = ade4*, *missing = ignore* and *cutoff = ignore*. The AMOVA was run through a dependency R package ADE4 v1.7-13^34,35^. Significance was assessed using a randomization test (***randtest***), based on 100,000 repetitions.

The population structure was analysed by applying the multivariate statistical method of Discriminant Analysis of Principal Components (DAPC), which identifies and describes clusters of genetically related individuals (***dapc***; ADEGENET v2.1.1^36,37^). This method also enabled the assignment of individuals to clusters and the contribution of individual alleles to population structuring, thereby characterizing population subdivision. Individual assignment utilized the true sampling locations as clusters (SCI priors given). When ***dapc*** was run, 30 axes were retained in the principal component analysis step (PCA; *n.pca = 30; n.pca* number was determined by cross-validation - ***xvalDapc***) and two axes were retained in the discriminant analysis (DA; *n.da = 2*) step.

A PCA was also performed to ensure the *de novo* DAPC population structure reflected an accurate representation of the overall population structure, despite migration rates. It has been found that when migration rates are high and groups are described *de novo* using a DAPC there may be substantial inaccuracy^38^. ***tab*** was used to standardise data as frequencies before using ***dudi.pca*** (ADEGENET and ADE4, respectively). A barplot was used to ensure the number of axes retained (*nf =2*; ***dudi.pca***) would contain the greatest biological signal.

## Spatial structure

### Migration rates

DIVERSITY v1.9.90^39,40^ was used to test and understand the influence that ocean currents have had on suspected directional asymmetric migration pattern among populations, and ultimately direction of gene flow. Patterns of allelic differentiation were used to estimate asymmetric relative rates of migration (***divMigrate***; DIVERSITY^40^). G_ST_^41^ was the statistical parameter selected to estimate the relative migration between populations. The mean relative migration rates and 95% confidence intervals were calculated using 10,000 bootstrap iterations.

### Particle modelling

Modelling of the movement of particles (i.e., proxy larvae) was undertaken with the open source particle tracking framework OPENDRIFT v1.0^42^ driven by the most sophisticated 3-dimensional hydrodynamic model for New Zealand’s ocean region (Moana Project New Zealand backbone v1.9^43^). This model is based on, and calibrated against, more than 25 years of detailed hydrodynamic hindcast data. The model uses high resolution bathymetry available for the shelf waters for the New Zealand region (i.e., General Bathymetric Chart of the Oceans (GEBCO) in combination with higher resolution locally-sourced data) and resolves 50 vertical sigma layers in the water column. The particle tracking hydrodynamic model was used to simulate the dispersal of scampi larvae during their pelagic phase from the sampling locations and the potential inter-generational connectivity trends among the scampi sampling locations, as a proxy for genetic exchange, and assumes the availability of viable intermediate scampi habitat where arriving genetic material can establish and reproduce to further disperse the genetic material. Thus, the particle dispersal model was intentionally run over an extended period to account for temporal variability in hydrodynamic processes and capture dispersal patterns in an analogous fashion to that of genetic connectivity estimates, as it is unlikely that seasonal changes would substantially change mean characteristics of circulation^44^.

The model domain covers New Zealand’s Exclusive Economic Zone (model domain coordinates: 161.03°, -31.03°; 184.97°, -31.03°; 161.03°, -51.98°; 184.97°, -51.98°) and places the open boundaries far enough to fully encompass the TF to the north and the STF to the south whilst also maintaining practicalities of the computational cost for the provision of high resolution models of this scale. The model operates on a temporal resolution of 24 hours and includes hourly atmospheric forcing and spectral tidal forcing at the model boundaries. Due to the poor swimming abilities of scampi throughout their larval development^45,46^ they were treated as passive particles in the model, moved by the 3-dimensional hydrodynamic forces generated from the model and a diffusion coefficient.

We selected particle tracking parameters based on previous hydrodynamic modelling of invertebrate larval dispersal in New Zealand^47-49^. A total of 146,100 particles were released on the surface over the course of the simulation, and their movements tracked to estimate the average multi-generational connectivity between SCIs. One hundred representative particles were released from evenly spaced locations within each sample area on each release step. Equal numbers of particles were released every 6 hours between 1 January 2008 and 31 December 2008 to ensure particles were seeded throughout the year to best capture the mean direction of particle movement. Particles were released over a year as scampi are presumed to release eggs year round, as berried females are captured throughout the year. The sample area corner coordinates were determined by using central trawl coordinates calculated from each of the five scampi sampling locations (Table S1) and then calculating coordinates that were 30 km north/south and 30 km east/west from the central coordinates, thus encapsulating the trawl area as best as possible. Particles were tracked using a modified version of the ***PelagicEgg*** tracking submodel included with OPENDRIFT until they met one of three conditions (1) the particles left the model domain, (2) they reached one of the other release sample areas, or (3) until 31 December 2017. The model operated with an internal timestep of 15 minutes and particle locations were recorded every seven days. A horizontal diffusion coefficient of 0.18 m^2^ s^-1^ was included to account for sub grid scale diffusion based on the equations of Okubo and Ebbesmeyer (1976)^50^. The raw particle modelling data was extracted using NCDF4 v1.17^51^. The data was used in several ways, (1) to extract the data pertaining to that of the pelagic larval phase per season (December to February – summer; March to May – Autumn; June to August – Winter; September to November – Spring), (2) to track the movement of the particles which could then provide details of the approximate number of generations needed for connectivity among dispersed locations, and (3) enumerating connectivity by calculating the total number of particles arriving at each SCI sample area from each source sample area over the modelling period (i.e., source-sink dynamics).

Our use of hydrodynamic models for simulating larval dispersal draws on well-established research for a wide range of marine species ^e.g.,52-55^. However, our approach to the application of the modelling differs in that we are interested in multigenerational connectivity which is consistent with the biological process for migration of genes within a wide ranging population, and it ensured that inter-annual variability in hydrodynamic processes were incorporated into the modelled outcomes, whilst also ensuring there was enough time for particle dispersal to assess possible long distance multigenerational dispersal.

# References

1 Ogilvie, S. *et al.* Mātauranga Māori driving innovation in the New Zealand scampi fishery. *New Zealand Journal of Marine and Freshwater Research* **52**, 590-602, doi:10.1080/00288330.2018.1532441 (2018).

2 Peterson, B. K., Weber, J. N., Kay, E. H., Fisher, H. S. & Hoekstra, H. E. Double digest RADseq: An inexpensive method for *de novo* SNP discovery and genotyping in model and non-model species. *PLoS ONE* **7**, e37135, doi:10.1371/journal.pone.0037135 (2012).

3 Elshire, R. J. *et al.* A robust, simple genotyping-by-sequencing (GBS) approach for high diversity species. *PLoS ONE* **6**, e19379, doi:10.1371/journal.pone.0019379 (2011).

4 Andrews, S. FastQC: A quality control tool for high throughput sequence data v. 0.11.7 (Babraham Bioinformatics, 2010). <http://www.bioinformatics.babraham.ac.uk/projects/fastqc>

5 Rochette, N. C., Rivera-Colón, A. G. & Catchen, J. M. Stacks 2: Analytical methods for paired-end sequencing improve RADseq-based population genomics. *Molecular Ecology* **28**, 4737-4754, doi:10.1111/mec.15253 (2019).

6 Danecek, P. *et al.* The variant call format and VCFtools. *Bioinformatics* **27**, 2156-2158, doi:10.1093/bioinformatics/btr330 (2011).

7 R Core Team. R: A language and environment for statistical computing v. 4.1.0 (R Studio v1.4.1106) (R Foundation for Statistical Computing, Vienna, Austria, 2021). <https://www.R-project.org/>

8 Díaz-Arce, N. & Rodríguez-Ezpeleta, N. Selecting RAD-seq data analysis parameters for population genetics: The more the better? *Frontiers in Genetics* **10**, 533, doi:10.3389/fgene.2019.00533 (2019).

9 Potapov, V. & Ong, J. L. Examining sources of error in PCR by single-molecule sequencing. *PLoS ONE* **12**, e0169774, doi:10.1371/journal.pone.0169774 (2017).

10 Waples, R. S. Testing for Hardy–Weinberg Proportions: Have We Lost the Plot? *Journal of Heredity* **106**, 1-19, doi:10.1093/jhered/esu062 (2015).

11 Pearman, W. S., Urban, L. & Alexander, A. Commonly used Hardy-Weinberg equilibrium filtering schemes impact population structure inferences using RADseq data. *bioRxiv*, 2021.2006.2015.448615, doi:10.1101/2021.06.15.448615 (2021).

12 Hendricks, S. *et al.* Recent advances in conservation and population genomics data analysis. *Evolutionary Applications* **11**, 1197-1211, doi:10.1111/eva.12659 (2018).

13 Luikart, G., England, P. R., Tallmon, D., Jordan, S. & Taberlet, P. The power and promise of population genomics: From genotyping to genome typing. *Nature Reviews Genetics* **4**, 981-994, doi:10.1038/nrg1226 (2003).

14 Pérez-Figueroa, A., García-Pereira, M. J., Saura, M., Rolán-Alvarez, E. & Caballero, A. Comparing three different methods to detect selective loci using dominant markers. *Journal of Evolutionary Biology* **23**, 2267-2276, doi:10.1111/j.1420-9101.2010.02093.x (2010).

15 Benestan, L. *et al.* Seascape genomics provides evidence for thermal adaptation and current-mediated population structure in American lobster (*Homarus americanus*). *Molecular Ecology* **25**, 5073-5092, doi:10.1111/mec.13811 (2016).

16 Narum, S. R. & Hess, J. E. Comparison of F_ST_ outlier tests for SNP loci under selection. *Molecular Ecology Resources* **11**, 184-194, doi:10.1111/j.1755-0998.2011.02987.x (2011).

17 Whitlock, M. C. & Lotterhos, K. E. Reliable detection of loci responsible for local adaptation: Inference of a null model through trimming the distribution of F_ST_. *The American Naturalist* **186**, S24-S36, doi:10.1086/682949 (2015).

18 Foll, M. & Gaggiotti, O. A genome-scan method to identify selected loci appropriate for both dominant and codominant markers: A Bayesian perspective. *Genetics* **180**, 977-993, doi:10.1534/genetics.108.092221 (2008).

19 Beaumont, M. A. & Balding, D. J. Identifying adaptive genetic divergence among populations from genome scans. *Molecular Ecology* **13**, 969-980, doi:10.1111/j.1365-294X.2004.02125.x (2004).

20 Weir, B. S. & Cockerham, C. C. Estimating F-statistics for the analysis of population structure. *Evolution* **38**, 1358-1370, doi:10.2307/2408641 (1984).

21 Frichot, E. & François, O. LEA: An R package for landscape and ecological association studies. *Methods in Ecology and Evolution* **6**, 925-929, doi:10.1111/2041-210x.12382 (2015).

22 Frichot, E., Mathieu, F., Trouillon, T., Bouchard, G. & François, O. Fast and efficient estimation of individual ancestry coefficients. *Genetics* **196**, 973-983, doi:10.1534/genetics.113.160572 (2014).

23 Martins, H., Caye, K., Luu, K., Blum, M. G. B. & François, O. Identifying outlier loci in admixed and in continuous populations using ancestral population differentiation statistics. *Molecular Ecology* **25**, 5029-5042, doi:10.1111/mec.13822 (2016).

24 Storey, J. D., Bass, A. J., Dabney, A. & Robinson, D. qvalue: Q-value estimation for false discovery rate control v. 2.15.0 (Bioconductor, 2017). <https://doi.org/10.18129/B9.bioc.qvalue>

25 Benson, D. A. *et al.* GenBank. *Nucleic Acids Research* **41**, D36-D42, doi:10.1093/nar/gks1195 (2013).

26 Morgulis, A. *et al.* Database indexing for production MegaBLAST searches. *Bioinformatics* **24**, 1757-1764, doi:10.1093/bioinformatics/btn322 (2008).

27 Gosselin, T. radiator: RADseq data exploration, manipulation and visualization using R v. 1.1.2 (GitHub, 2019). <https://thierrygosselin.github.io/radiator/>

28 Goudet, J. & Jombart, T. hierfstat: Estimation and tests of hierarchical F-statistics v. 0.04-22 (Comprehensive R Archive Network (CRAN), 2015). <https://CRAN.R-project.org/package=hierfstat>

29 Nei, M. Molecular evolutionary genetics. *Columbia University Press* (1987).

30 Nei, M. & Chesser, R. K. Estimation of fixation indices and gene diversities. *Annals of Human Genetics* **47**, 253-259, doi:10.1111/j.1469-1809.1983.tb00993.x (1983).

31 Archer, F. I., Adams, P. E. & Schneiders, B. B. stratag: An R package for manipulating, summarizing and analysing population genetic data. *Molecular Ecology Resources* **17**, 5-11, doi:10.1111/1755-0998.12559 (2017).

32 Kamvar, Z. N., Tabima, J. F. & Grünwald, N. J. Poppr: An R package for genetic analysis of populations with clonal, partially clonal, and/or sexual reproduction. *PeerJ* **2**, e281, doi:10.7717/peerj.281 (2014).

33 Kamvar, Z. N., Brooks, J. C. & Grünwald, N. J. Novel R tools for analysis of genome-wide population genetic data with emphasis on clonality. *Frontiers in Genetics* **6**, 208, doi:10.3389/fgene.2015.00208 (2015).

34 Bougeard, S. & Dray, S. Supervised multiblock analysis in R with the ade4 package. *Journal of Statistical Software* **86**, 17, doi:10.18637/jss.v086.i01 (2018).

35 Dray, S. & Dufour, A.-B. The ade4 package: Implementing the duality diagram for ecologists. *Journal of Statistical Software* **22**, 1-20, doi:10.18637/jss.v022.i04 (2007).

36 Jombart, T. *adegenet*: A R package for the multivariate analysis of genetic markers. *Bioinformatics* **24**, 1403-1405, doi:10.1093/bioinformatics/btn129 (2008).

37 Jombart, T. & Ahmed, I. *adegenet 1.3-1*: New tools for the analysis of genome-wide SNP data. *Bioinformatics* **27**, 3070-3071, doi:10.1093/bioinformatics/btr521 (2011).

38 Miller, J. M., Cullingham, C. I. & Peery, R. M. The influence of a priori grouping on inference of genetic clusters: simulation study and literature review of the DAPC method. *Heredity* **125**, 269-280, doi:10.1038/s41437-020-0348-2 (2020).

39 Keenan, K., McGinnity, P., Cross, T. F., Crozier, W. W. & Prodöhl, P. A. diveRsity: An R package for the estimation and exploration of population genetics parameters and their associated errors. *Methods in Ecology and Evolution* **4**, 782-788, doi:10.1111/2041-210x.12067 (2013).

40 Sundqvist, L., Keenan, K., Zackrisson, M., Prodöhl, P. & Kleinhans, D. Directional genetic differentiation and relative migration. *Ecology and Evolution* **6**, 3461-3475, doi:10.1002/ece3.2096 (2016).

41 Nei, M. Analysis of gene diversity in subdivided populations. *Proceedings of the National Academy of Sciences* **70**, 3321-3323, doi:10.1073/pnas.70.12.3321 (1973).

42 Dagestad, K. F., Röhrs, J., Breivik, Ø. & Ådlandsvik, B. OpenDrift v1.0: a generic framework for trajectory modelling. *Geoscientific Model Development* **11**, 1405-1420, doi:10.5194/gmd-11-1405-2018 (2018).

43 de Souza, J. M. A. C. *et al.* Moana Ocean Hindcast – a 25+ years simulation for New Zealand Waters using the ROMS v3.9 model. *EGUsphere* **2022**, 1-34, doi:10.5194/egusphere-2022-41 (2022).

44 Bracco, A., Liu, G., Galaska, M. P., Quattrini, A. M. & Herrera, S. Integrating physical circulation models and genetic approaches to investigate population connectivity in deep-sea corals. *Journal of Marine Systems* **198**, 103189, doi:10.1016/j.jmarsys.2019.103189 (2019).

45 Jeffs, A., Daniels, C. & Heasman, K. in *Fisheries and Aquaculture: Natural History of Crustacea* Vol. 9 (eds G. Lovrich & M. Thiel) 285-311 (Oxford University Press, 2020).

46 Heasman, K. G. & Jeffs, A. G. Fecundity and potential juvenile production for aquaculture of the New Zealand scampi, *Metanephrops challengeri* (Balss, 1914) (Decapoda: Nephropidae). *Aquaculture* **511**, 634184, doi:10.1016/j.aquaculture.2019.05.069 (2019).

47 Lundquist, C. J., Oldman, J. W. & Lewis, M. J. Predicting suitability of cockle *Austrovenus stutchburyi* restoration sites using hydrodynamic models of larval dispersal. *New Zealand Journal of Marine and Freshwater Research* **43**, 735-748, doi:10.1080/00288330909510038 (2009).

48 Lundquist, C. J., Thrush, S. F., Oldman, J. W. & Senior, A. K. Limited transport and recolonization potential in shallow tidal estuaries. *Limnology and Oceanography* **49**, 386-395, doi:10.4319/lo.2004.49.2.0386 (2004).

49 Norrie, C., Dunphy, B., Roughan, M., Weppe, S. & Lundquist, C. Spill-over from aquaculture may provide a larval subsidy for the restoration of mussel reefs. *Aquaculture Environment Interactions* **12**, 231-249 (2020).

50 Okubo, A. & Ebbesmeyer, C. C. Determination of vorticity, divergence, and deformation rates from analysis of drogue observations. *Deep Sea Research and Oceanographic Abstracts* **23**, 349-352, doi:10.1016/0011-7471(76)90875-5 (1976).

51 Pierce, D. ncdf4: Interface to unidata netCDF (version 4 or earlier) format data files v. 1.17 (Comprehensive R Archive Network (CRAN), 2019). <https://CRAN.R-project.org/package=ncdf4>

52 Coelho, S. C. C., Gherardi, D. F. M., Gouveia, M. B. & Kitahara, M. V. Western boundary currents drive sun-coral (*Tubastraea* spp.) coastal invasion from oil platforms. *Scientific Reports* **12**, 5286, doi:10.1038/s41598-022-09269-8 (2022).

53 Demmer, J. *et al.* The role of wind in controlling the connectivity of blue mussels (*Mytilus edulis L.*) populations. *Movement Ecology* **10**, 3, doi:10.1186/s40462-022-00301-0 (2022).

54 Atalah, J., South, P. M., Briscoe, D. K. & Vennell, R. Inferring parental areas of juvenile mussels using hydrodynamic modelling. *Aquaculture* **555**, 738227, doi:10.1016/j.aquaculture.2022.738227 (2022).

55 McGeady, R., Lordan, C. & Power, A. M. Long-term interannual variability in larval dispersal and connectivity of the Norway lobster (*Nephrops norvegicus*) around Ireland: When supply-side matters. *Fisheries Oceanography* **31**, 255-270, doi:10.1111/fog.12576 (2022).
